# Nanoparticles and intracellular applications of surface-enhanced Raman spectroscopy

**DOI:** 10.1039/c6an01003b

**Published:** 2016-07-19

**Authors:** Jack Taylor, Anna Huefner, Li Li, Jonathan Wingfield, Sumeet Mahajan

**Affiliations:** a Department of Chemistry and Institute of Life Sciences (IfLS) , University of Southampton , SO17 1BJ , UK . Email: s.mahajan@soton.ac.uk; b Sector for Biological and Soft Systems , Cavendish Laboratory , Department of Physics , University of Cambridge , 19 JJ Thomson Avenue , Cambridge , CB3 0HE , UK; c Discovery Sciences , Screening and Compound Management , AstraZeneca , Unit 310 - Darwin Building , Cambridge Science Park , Milton Road , Cambridge , CB4 0WG , UK

## Abstract

Surface-enhanced Raman spectrocopy (SERS) offers ultrasensitive vibrational fingerprinting at the nanoscale. Its non-destructive nature affords an ideal tool for interrogation of the intracellular environment, detecting the localisation of biomolecules, delivery and monitoring of therapeutics and for characterisation of complex cellular processes at the molecular level. Innovations in nanotechnology have produced a wide selection of novel, purpose-built plasmonic nanostructures capable of high SERS enhancement for intracellular probing while microfluidic technologies are being utilised to reproducibly synthesise nanoparticle (NP) probes at large scale and in high throughput. Sophisticated multivariate analysis techniques unlock the wealth of previously unattainable biomolecular information contained within large and multidimensional SERS datasets. Thus, with suitable combination of experimental techniques and analytics, SERS boasts enormous potential for cell based assays and to expand our understanding of the intracellular environment. In this review we trace the pathway to utilisation of nanomaterials for intracellular SERS. Thus we review and assess nanoparticle synthesis methods, their toxicity and cell interactions before presenting significant developments in intracellular SERS methodologies and how identified challenges can be addressed.

## Abstract 2

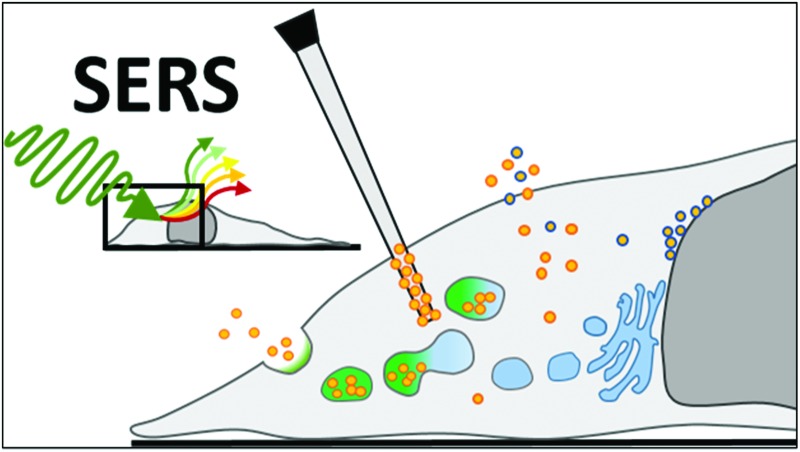
Surface-enhanced Raman spectroscopy offers ultra-sensitive vibrational fingerprinting within biological cells.

## Introduction

Nanostructures such as carbon nanotubes, graphene, quantum dots, and nanomaterials made out of metals, semiconductors, non-metallic oxides and polymers have been developed for numerous biomedical applications including targeted delivery of drugs and genes, bioimaging, biosensing, and cancer treatment. Of particular interest are plasmonic nanoparticles (NPs), primarily of gold (Au) and silver (Ag), owing to their unique optical properties which allow intense scattering of light to achieve quantification, localisation and therefore imaging of biological systems^1^ down to the molecular level.

Surface-enhanced Raman spectroscopy (SERS), potentiated by noble metal nanostructures, was first observed in 1973 and subsequently verified in 1977, when the spontaneous Raman signal of adsorbed pyridine was easily measured at a roughened silver electrode.^2–4^ The heightened intensities observed in SERS relative to spontaneous Raman spectroscopy are primarily due to the enhanced electric fields produced by conductance electrons at nanomaterial surfaces, which undergo collective oscillations known as surface plasmons. Combination of this electromagnetic mechanism with additional pathways such as charge transfer and chemisorption induced resonance Raman effects result in enhancement by factors of 10^6^–10^10^ in SERS^5,6^ over spontaneous Raman spectroscopy. Such enhancement is crucial to studies of intact and living cells as the concentrations of biomolecules inside cells are typically of the order of nM. It allows fine spectral details to be observed without interference from the vibrational peaks of H_2_O observed in IR spectroscopy. (Surface-enhanced) Raman spectroscopy also proves advantageous as it is a non-destructive and label-free tool with simple or no preparation of samples, utilising an increased depth of penetration by NIR radiation. Currently, fluorescence imaging is commonplace and benefits as an intracellular technique from large intrinsic signals, availability of a wide range of labels (including a large palette of fluorescent proteins which can be incorporated endogenously through genetic modification) and the ability to tune the response of labels to analytes or pH.^7^ However, it lacks the specificity of information provided by SERS, as only a finite number of dyes can be simultaneously employed for probing the desired environment due to spectral overlap. Such tagging of molecules can also perturb the natural, molecular-level progression of biological pathways being analysed.^8^ It is worth noting that prolonged exposure to nanoparticles can also play an active role in mediating biological effects.^9,10^ However, fluorescence has further limitations that signals get photobleached over time^8^ compared to Raman-based techniques. Given that SERS has been shown to possess single molecule sensitivity^11–13^ and can be comparable or more sensitive than fluorescence for biological assays^14,15^ it offers numerous advantages and complimentary information for intracellular analysis.

For successful cellular investigations by SERS, however, the selection of suitable NPs is essential, which must overcome issues such as internalisation and toxicity while maintaining desired optical properties. For *in cellulo* studies, particle diameter must be small enough to penetrate the intracellular matrix yet larger than 15 nm to achieve SERS enhancement.^16^ Spherical AgNPs exhibit stronger plasmonic fields than those of Au, especially in the visible region of the electromagnetic spectrum owing to the partial Au plasmon band overlap with its interband electronic transitions. Notwithstanding this, AuNPs are more widely applied in biological studies due to their well established and controlled methods of synthesis along with good biocompatibility and chemical stability. The ability to track and detect plasmonic NPs using various analytical tools, especially their localised surface plasmon resonance bands, which can be synthetically tuned into the near infrared region (the optical transparency window for biological tissues), is an added advantage. Facile surface chemistry allows for easy surface functionalisation, affording not only the binding of specific delivery peptides, but also other applications such as artificial antibodies with binding affinities precisely tuned by varying the density of surface bound ligands. The ability to shield unstable drugs or poorly soluble imaging contrast agents to facilitate their delivery to otherwise inaccessible regions of the body is augmented by AuNPs’ multivalent nature.^17^

From the above it can thus be seen that the type of nanoparticles, choice of their surface chemistry and consequent interaction with cells (uptake, toxicity) can be critical to their utilisation for intracellular SERS. Through this review we therefore aim to provide an insight into all aspects involving intracellular SERS sensing and imaging. We trace all the steps involved in the use of nanostructures for intracellular SERS, starting with an evaluation of the main synthetic methods and different types of nanoparticles. While spherical AuNPs are currently the most widely used and understood, AgNPs have been used and alternative structures are becoming popular and will be discussed where relevant. This will be followed by an overview of crucial NP–cell interactions. Physical and diffusive routes of particle internalisation are examined along with biocompatibility factors such as tunability of surface properties for internalisation and resulting toxicity. The two prevalent approaches, the SERS-reporter and reporter-free approaches, to intracellular SERS are then presented. State-of-the-art and recent significant studies are discussed and the potential of intracellular SERS for numerous applications in life sciences, therapeutics and drug discovery is discussed.

## Nanoparticle synthesis

The earliest description of obtaining colloidal Au can be traced to ancient Chinese, Arabian, and Indian treatises dating back to as early as 4–5th BCE wherein it was utilised mostly for medicinal purposes.^18,19^ However, it was Michael Faraday's recognition that the properties of pure colloidal (or ‘activated’) Au, following its synthesis by reduction of Au chloride by phosphorus in the presence of carbon disulphide as a stabiliser, were due to its minute size,^20^ which attracted renewed scientific attention. With the wide availability of nanoscale characterisation tools, the design of AuNPs with precisely controlled sizes and shapes has since been comprehensively developed.

**Fig. 1:**
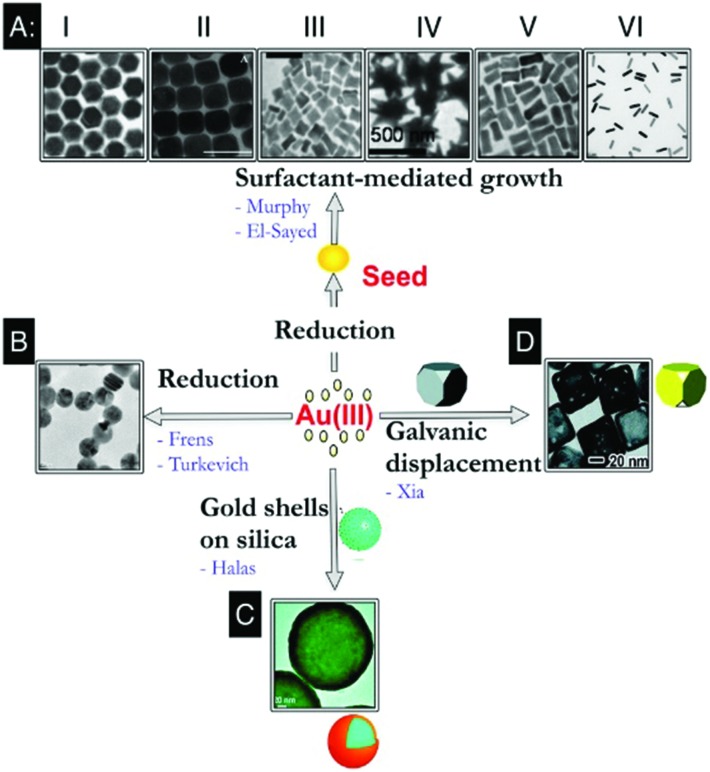
Representative synthetic approaches to prepare AuNPs with controllable size and shape. (A) Surfactant-mediated solution-based growth method to prepare AuNPs with hexagonal (A, I), cubic (A, II), rectangular (A, III), star (A, IV), dog bone (A, V), and rod (A, VI) shapes. (B) Citrate-mediated growth method to prepare Au nanospheres *via* direct reduction of Au ions. (C) Sequential growth method to prepare Au nanoshell on silica. (D) Galvanic replacement reaction on the surface of Ag nanocubes to prepare porous/hollow Au nanocages. Reprinted with permission from Alkilany *et al.*, *Acc. Soc. Res.*, 2013, **46**, 650–661. Copyright (2013) American Chemical Society. Images in A and D are reprinted with permission from ref. 25 and 35 respectively; Copyright (2004, 2011, respectively) American Chemical Society. Images in C is reprinted with permission from Wang *et al.*, *Acc. Chem. Res.*, 2007, **40**, 5362; Copyright (2007) American Chemical Society.

Depending upon the desired application, a variety of AuNP structures can be synthesised, ranging from nanospheres to nanowires, nanorods (NRs) with high aspect ratios, nanocages with a hollow interior and top down deposited nanochips. The size of AuNPs can also be precisely tuned from a few to hundreds of nm.^1,17^ Fig. 1 summarises several representative synthetic methods for preparation of AuNPs with controllable size and shape. Monodisperse Au nanospheres with 16–250 nm diameters can be synthesized *via* a citrate-mediated growth method, which was first demonstrated by Turkevich *et al.* in 1951 ^21^ and later systematically studied and improved by Frens^22^ (Fig. 1B). A “necklace breaking” mechanism is typically involved in which small nuclei (5 nm in diameter) first assemble into chains, which interconnect and grow thicker. AuNPs eventually dissociate from these to generate monodisperse Au nanospheres.^23^ Murphy *et al.* later established a surfactant-mediated wet chemical growth method, allowing variation of the morphology and dimensions of AuNPs by adjusting reaction conditions^24,25^ (Fig. 1A). This method mixes pre-synthesised single crystal Au seeds with aqueous growth solutions containing appropriate quantities of cetyltrimethylammonium bromide (CTAB), HAuCl_4_, ascorbic acid, and AgNO_3_. The fine control of NP morphology, such as NRs,^26^ nanocubes,^27,28^ nanotriangles^29^ and nanostars^30–32^ can be achieved by systematically varying the synthetic parameters. Generally, most wet-chemistry methods for the synthesis of NPs follow a similar protocol which is still widely used, in which solvated metal salt is reduced in the presence of stabilising surfactants and/or ligands. However, it is to be noted that NP growth and the subsequent control of NP morphology and size is a complex process strongly influenced by reaction kinetics, thermodynamics and stabilisation effects. Therefore, small changes in reaction parameters can often result in very different products.

Wet-chemistry methods such as the above provide AuNPs with a whole library of morphologies and sizes available not only for various biomedical applications, but also as building blocks for fabrication of further nanostructures. For example, Au nanoshells have attracted much attention due to their interesting plasmonic properties and exciting applications. Halas *et al.* developed a sequential growth method for achieving better control over monodispersity of both the silica core and the Au nanoshell with both high purity and high yield.^33^ The ultrasmall (1–2 nm in diameter) AuNPs were first anchored onto silica NP cores, followed by further reduction to form a complete Au shell layer (Fig. 1C). The thickness of Au shell layers is precisely tuned by varying the amount of Au deposited on the surface of the silica-core NPs. Silica-shell Au core NPs have also been recently developed for SERS based nanosensors. Tian *et al.* designed AuNPs coated with an ultrathin (2 nm) shell of silica or alumina,^34^ preventing uncontrolled AuNP aggregation while retaining strong optical enhancement. In addition, the chemically inert and fully enclosed shell layer allows particles to adjust its conformation to the diverse contours of non-flat samples such as single-crystal edges and biological cells. Au nanocages developed by Xia *et al.* possess unique cargo-holding hollow structures in addition to attractive optical properties, thus are useful for many biomedical applications, including bioimaging, cancer diagnosis, photothermal therapy, and drug delivery^35^ (Fig. 1D). In this approach, porous/hollow Au nanocages were obtained *via* a previously established galvanic replacement reaction^36–38^ of Ag nanocubes with HAuCl_4_ at their surface.

Most syntheses reviewed here provide fairly simple and low cost, yet high yield synthetic routes for the fabrication of AuNPs with different morphologies and tunable sizes. However, most of them are conducted in a batch format, usually producing small quantities (<1 g) with multiple steps and poor inter-batch reproducibility. Simply scaling up the reactant volumes does not necessarily result in the same reaction scheme, because nucleation and growth of colloids are very complex processes that are extremely sensitive to experimental conditions. Implementing continuous and automated NP production processes is therefore desirable to facilitate scale up and improve homogeneity.

Xia *et al.* showed that by using microfluidic droplet reactors, continuous-flow mass production of a number of noble-metal NPs with controlled sizes and shapes can be achieved.^39^ In this method, microlitre-sized droplets containing various reagents were continuously generated in a fluidic device fabricated by assembling silica capillaries inside polymer tubes. Microfluidic techniques are operated at a steady state, offering superior control over reaction conditions such as reagent addition, mixing, reaction time and temperature. NP size depends upon relative reaction and mixing rates along with the extent of polydispersity, enhanced by the improved control over heat and mass transport which accompanies the technology.^39^ The approach also lends itself to accurate and rapid screening of conditions for quality control purposes.

Microfluidic AgNP synthesis has been performed in a continuous flow single-mode microwave reactor^40^ using a previously established ‘polyol process’.^41–43^ This reduction method of ionic, inorganic precursors dissolved in liquid polyols (polyhydroxy alcohols) utilises microwave irradiation to achieve more uniform heating, nucleation control and increased reaction rates. Thus AgNPs could be produced from a silver acetate substrate within a matter of seconds.^40^ The penetration depth of microwaves (a few centimetres) renders the scale-up of batch production impractical yet is still large enough to rationally design a continuous-flow synthesis system with high yields.

**Fig. 2:**
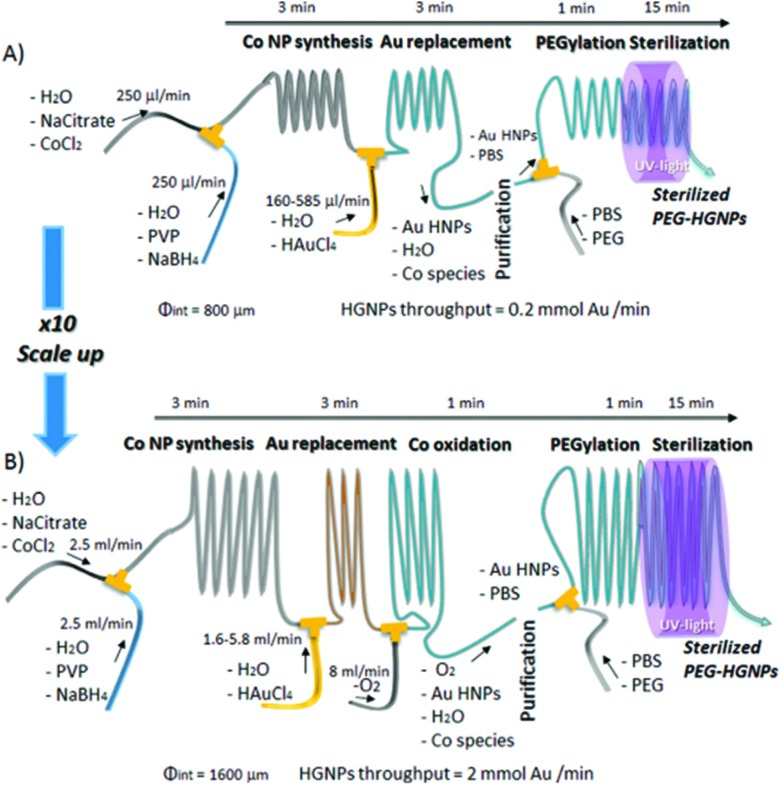
Experimental set up for the production of HGNPs produced from galvanic replacement of sacrificial Co templates, including stages PEG functionalisation and sterilisation on a singular platform, scaled up for high throughput. Reprinted with permission from L. Gomez, V. Sebastian, S. Irusta, A. Ibarra, M. Arruebo and J. Santamaria, *Lab Chip*, 2014, **14**, 325–332. Copyright (2014) Lab Chip.

Similarly, hollow gold nanoparticles (HGNPs) particularly require strict dimensional control within large-scale production, unachievable by batch processes. This is because the ratio of shell diameter to thickness determines the peak position of its surface plasmon resonance (SPR), while uniformity controls SPR bandwidth. High quality HGNPs were produced from galvanic replacement of sacrificial Co templates, including stages for poly-ethylene glycol (PEG) functionalisation and sterilisation on a singular platform with rational use of reactants and no need for time consuming purification steps by the scheme depicted in Fig. 2. In this regard, continuous microfluidic systems must be considered as a key tool in the future of large-scale and green routes for NP synthesis.^44^

Aside from the capability for scaling-up, modern synthetic chemistry is required to be sustainable. Another ‘green’ synthetic route for AuNP production lies in biosynthesis, with methods not requiring the high-energy input of irradiation based methods. An approach presented by Sathishkumar *et al.* utilises extract from commercially available *Illicium verum* (star anise, SA) in an effective one-step and economical synthesis of biocompatible AuNPs at room temperature. Although boiling is required for extraction, biologically synthesised AuNPs exhibited 20× and 5× fold toxicity reduction in A549 cells compared to those produced synthetically *via* sodium borohydrate and citrate reduction mechanisms respectively. This was attributed to the protective capping of NPs by polyphenols present in SA extract, with bio-AuNP exposed cells displaying reduced caspase levels, a key protein in the mechanism of apoptosis.^45^ AuNPs have been biosynthesised using the fruit extract of *Couroupita guianensis* (cannonball fruit) as a bio-reducing agent of Au^3+^ to yield anisotropic face centred cubic crystalline structured particles of 26 ± 11 nm diameter, with high stability and purity.^46^ These NPs also possessed extraordinary antioxidant properties, as revealed by *in vitro* assays, and free radical scavenging capabilities making them ideally suited to nanomedicine. In a similar vein, AgNPs have also been extracellularly synthesised without surfactant or external energy by mixing silver solution with extracts from *Azadiracta indica* and *Zingiber officiale* (Neem and Ginger respectively).^47,48^ This synthesis produced physiologically stable, highly biocompatible AgNPs within 1–2 h compared to the 2–4 days required by microorganisms in *in vivo* NP production. Biosynthesised NPs produced by Potara *et al.* were used to probe C26 murine colon carcinoma cells *in vitro* to reveal subcellular components and the localisation of the AgNPs by confocal Raman spectroscopy and *K*-means clustering analysis.^48^ Further to the examples provided, a comprehensive review of green NP sources and their significance to cancer management and developing anti-cancer stem cell therapeutics through enhanced biocompatibility is presented by the Sreelekha and coworkers.^49^ As with traditional synthetic methods, the major limitation of biosyntheses lies in their batch production format, presenting difficulties in upscaling production. A lack of inter-batch reproducibility was also reported as a result of natural variation in the polyphenol content of SA, despite minimal variation in toxicity within each batch of product.^45^ Therefore, the classical trade-off between sustainable and large-scale industrial synthesis is also applicable to NP production, thus requiring further development.

## Nanoparticle–cell interactions

The interaction between living cells and any exogenous material is one of great complexity. The cellular matrix is a vastly complicated environment which plays host to a range of internal structures and sensitive biochemical pathways, possessing variety in cargo uptake processes and transport vesicles. Therefore, it is with caution that NPs are introduced to living cells for developing models of healthy *in vivo* cells, as mechanistic understanding of how physiochemical properties of NPs affect NP/cellular interactions is limited. Although AuNPs are generally considered non-toxic to live cells (discussed later), evidence exists that their prolonged presence within the intracellular matrix does perturb their biology and induces stress.^9,10^ SERS has been carried out using glass capillary probes decorated with AuNPs to detect proteins or metabolites both intra-^50–52^ and extracellularly^53^ to overcome this issue. This review however focusses on the more widely applied NP-based intracellular SERS approach, in which NPs can be applied to samples as a probe reagent for internalisation at the time of analysis so as to minimise the effects of prolonged exposure on cellular pathways and thus yield sizeable advantages over conventional labelling techniques.

### Cellular internalisation methods

The key to successful utilisation of NPs inside cells for SERS and other measurements is understanding and manipulating their mechanism of uptake and cellular distribution. Internalisation methods generally fall into three categories: involuntary delivery by physical methods, passive diffusion, and active (or voluntary) uptake.

The basis of physical insertion methods is the theory of applying a force to the cell in order to create localised membrane pores and increase cell permeability. Electroporation and microinjection achieve this with the application of electrical and physical force respectively.^54^ Microinjection has been of particular interest in single cell studies, providing tight control of dosage and timing of delivery but is highly sensitive as inappropriate use (injection pressure, location) can easily damage cellular components. Single 100 nm AuNPs have been delivered to mammalian cell nuclei by combination of optical tweezing and opto-injection,^55^ along with using laser irradiation to also assist NP injection of 15–30 nm AuNPs.^56^ Despite the tight control offered, the complexity of both procedure and instrumentation limits the technique which is also confined to larger NPs.^57^ The major advantage of NP uptake by passive diffusion is that internalised cargos enter the cytosol directly and are not required to escape from vesicles involved in endocytosis. Colloidal semiconductor core/shell quantum dots coated with the zwitterionic thiol ligand d-penicillamine are effectively similar to globular proteins in size and surface charge, and are internalised by HeLa cells *via* clathrin-mediated endocytosis.^58^ However, these were also shown to enter erythrocytes by passive diffusion (given their lack of endocytotic machinery) without formation of holes in the lipid bilayer.^59^ AuNPs of diameter ≤200 nm have also been observed to penetrate the bilayer of erythrocytes.^60^

The uptake mechanism of AgNPs in yeast cells was recently investigated, comparing entry by electroporation with diffusive approaches by TATHA2 peptide functionalised (facilitated) and citrate capped AgNPs for uptake by endocytosis.^54^ The TATHA2 is 1 to 20 amino acid sequence of the influenza A virus hemagglutinin protein (HA2) connected to a 10 amino acid cell permeable HIV Trans-Activator of Transcription (TAT) protein transduction domain (PTD). The TAT PTD binds to the cell surface and penetrates the membrane while the pH sensitive lipid membrane destabilising sequence of the HA2 domain facilitates endosomal escape and transduction of the fusion peptide. In the above work by Bhardwaj *et al.* ^54^ it was found that although yeast cells can tolerate high electroporation doses, severe damage was observed with the presence of AgNPs. Free diffusion of AgNPs resulted in poor uptake, internalisation and endosome entrapment whereas these parameters were rapid and large with TATHA2-AgNPs. A uniform intracellular distribution was also observed, which is a requirement for detection of ubiquitously distributed molecules in cell based biosensors. Conjugation of cell penetrating peptides (CPP) such as arginylglyclaspartic (RGD) or nuclear localisation signal peptides (NLS) to NPs also facilitate cellular uptake,^61–65^ as well as help guide probes out of the endolysosomal pathway as discussed under the section on ‘Manipulating interactions’.

Voluntary uptake of particles *via* endocytosis is by far the most commonly used internalisation strategy, whereby metallic species are taken up by the cell's intrinsic machinery. This is owing to the method's relative simplicity and minimal sample preparation as well as tunability by appropriate surface modification.^66^

### The endocytotic pathway

**Fig. 3:**
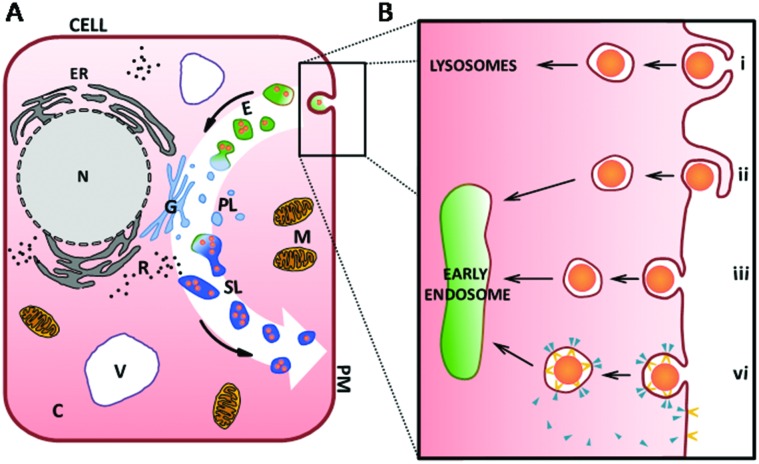
(A) A schematic of a cell highlighting different compartments. The endocytotic pathway is indicated by the white arrow in the background. After internalisation of the cargo into membrane-bound vesicles inside the cell (light-green, (B) for more details), these vesicles fuse to early endosomes and mature to late endosomes, here shown as green vesicles. Primary lysosomes (abbr. PL, here with light blue colouration) emerge from the Golgi apparatus (G) and merge with endosomes (E, green colouration) to form secondary lysosomes (SL, darker blue colouration). Passing through maturation and degradation processes, they get eventually exocytosed by the cell. (B) Detailed schematic showing the main mechanisms of endocytosis featuring phagocytosis (i), macropinocytosis (ii), non-specific absorptive endocytosis (iii) and receptor-mediated endocytosis (vi) involving receptor (yellow shape) and coating molecules (*e.g.* clathrin- and caveolin, blue triangle). Abbr.: cytoplasm (C), early and late endosomes (E), endoplasmic reticulum (ER), Golgi apparatus (G), mitochondria (M), nucleus (N), primary lysosome (PL), plasma membrane (PM), ribosome (R), secondary lysosome (SL), vacuole (V). Figure is not to scale.

For the intake of small proteins and ions into the cell, special transport channels enable their translocation across the cell membrane.^67^ For macromolecules and proteins which are too large to pass directly through the plasma membrane, the cell possesses different mechanisms for their intake from the environment. These intake mechanisms are generally referred to as endocytosis,^67^ the commencement of the vesicular endolysosomal pathway through the cell (white arrow in Fig. 3A).

In the first step of endocytosis, cargo for internalisation is engulfed by invagination, or pit formation, of the plasma membrane (black box in Fig. 3A). Pinching off from the plasma membrane, a small membrane-bound vesicle is formed which encapsulates the ingested molecule(s). Many routes of endocytic uptake into the cell exist. A particular intake pathway depends on the kind, size, shape and surface characteristics of the molecules (*e.g.* charge) as well as the specific cell type. Broadly, these pathways are divided into phagocytosis, macropinocytosis, endocytosis *via* non-coated vesicles and energy-dependent, receptor-mediated endocytosis^68–73^ (Fig. 3Bi–vi respectively).

There are a number of endocytotic mechanisms which are non-specific, absorptive and/or use processes that have not yet been identified (Fig. 3Biii). In contrast, receptor mediated-endocytosis (RME) has been studied extensively.^67^ RME is linked to the interaction of the receptor and the cargo to initiate the internalisation process *via* pit formation. Vesicles emerging from the plasma membrane usually have a size of 50–200 nm depending on the involved receptor and coating molecules (*e.g.* clathrin or caveolin). The receptor is initially internalised with the ligand and is gradually recycled to the plasma membrane (Fig. 3Bvi). Thus, this process is saturable and used for transmembranal signal transduction.^67^ Alongside with the disassembly of the coating molecules from the individual vesicles as shown in Fig. 3Bvi, these vesicles fuse to form early endosomes (light-green vesicles in Fig. 3).

Early endosomes are initially relatively small, have a mildly acidic pH and are known to be the main sorting station in the endosomal pathway. While intravesicular fluids are recycled, early endosomes fuse with other incoming cargos from different internalisation pathways (as shown in Fig. 3A and B) and undergo a maturation process developing into late endosomes (green vesicles in Fig. 3A).^71,74^ While this happens, the pH inside the vesicles decreases further. Late endosomes are bigger in size and their morphology changes during maturation.^71^ At the end of the endosomal pathway, late endosomes as well as phagosomes eventually fuse with primary lysosomes to form secondary lysosomes or phagolysosomes, containing components from both fusion partners.^71^ Hereafter, NP cargos can localise into various cellular compartments dependent on characteristics such as size and surface coating. Transport to the nuclear membrane, translocation across the nuclear membrane and rejection from the cell by exocytosis can all occur, although penetration into nuclear membrane is difficult to achieve with administered NPs clustering at the membrane or dispersing in the cytoplasm.^16,66,75^ The fused primary lysosomes emerge from the Golgi apparatus (Fig. 3A) and contain numerous enzymes (*e.g.* hydrolase) along with various macromolecules from other intracellular, catabolic processes and due to membrane turnover. These include molecules incorporated previously through autophagy.^71,76^ During the lysosomal pathway, most of the molecular digestion and degradation takes place while the pH ^77^ in these vesicles becomes more and more acidic. Subsequent dynamic changes of the molecular composition and the activity of enzymes inside the vesicles also result in modification of the encapsulated molecules.^67,69,78^ At the end of the lysosomal pathway, vesicles with indigestible material become residual bodies and are secreted from the cell *via* exocytosis.^24,76,79^ Different maturation and fusion steps within this whole pathway are colour-indicated in Fig. 3A.

The advantage of the uptake of plasmonic NPs by endocytosis is its simplicity, both in execution and sample preparation. The typical procedure following particle synthesis consists of an incubation period of cells with NPs suspended in culture medium before washing and SERS investigation. Tunability is afforded by altering NP properties such as size, shape and surface coating, which can be manipulated to localise SERS nanoprobes to desired cellular locations or access or escape specific pathways and transport vesicles.

### Manipulating interactions

It is of great importance to understand and quantify the intracellular uptake of AuNPs. Extensive studies have shown that many factors, including size, shape, surface coating, concentration of NPs, aggregation state of NPs, the type of cell, the type of culture media and the exposure conditions play important roles in their biological interactions.^80^ With innovations in nanotechnology, a library of fit-for-purpose AuNPs with different physicochemical properties has been established.^5,81–88^

A straightforward parameter to manipulate during synthesis is NP size (diameter). With the intention to carry out intracellular SERS experimentation, several factors contribute to identifying the optimal NP diameter. AuNPs larger than 15 nm have a sufficiently large scattering cross-section and exhibit plasmonic activity to provide the required optical contrast and electric field enhancement, which must be considered when utilizing optical based analytical tools for the localization of AuNPs inside cells.^89^ Additionally, a NP must also be efficiently internalised by the cell. Studies have shown that the optimal diameter for endocytotic uptake of AuNPs is around 50 nm; although this depends on other factors previously mentioned. Size dependence of uptake is associated with ‘wrapping time’, the numerically determined time required for elevation of membrane receptor density to achieve complete invagination of the NP, with all of its surface area in contact with membrane. This is affected by factors including ratio of adhesion, membrane stretching and membrane bending. Optimal values of wrapping time have been calculated as 2–58 s for three dimensional NPs of diameters 54–60 nm.^77^ Practically, HeLa cells in Dulbecco medium exhibited greater uptake of 50 nm AuNPs compared to sizes of 14, 30, 74 and 100 nm, showing agreement with theoretical simulations.^79,80^ It was shown that 55 nm AuNPs had the fastest wrapping time, with RME receptor binding of a NP producing enough free energy to drive it into the cell. This free energy is reduced in NPs of diameter <50 nm, which are therefore required to cluster together in order to achieve internalisation. 14 nm AuNPs are thus required to cluster with at least 5 other particles for internalisation. Conversely, wrapping times of large NPs are increased because more RME receptors are required to bind their larger surface area and generate sufficient free energy for internalisation. Consequently, the number of AuNPs entering the cell in transport vesicles is reduced; with uptake rate limited by the speed of diffusion of extra receptors to the site of invagination.^77,79^ Hence, most intracellular SERS studies conducted utilise spherical AuNPs in the diameter range 40–60 nm.

**Fig. 4:**
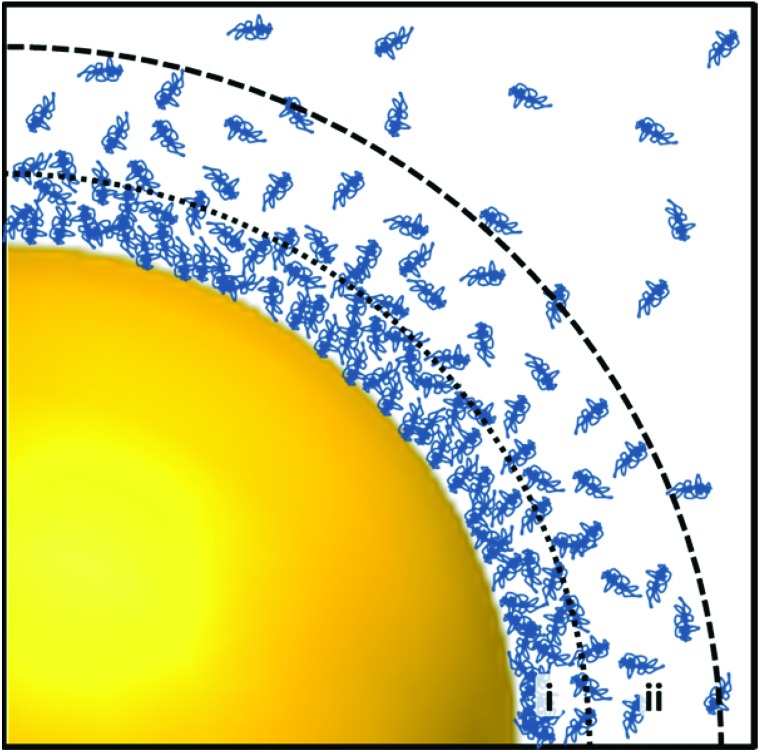
Illustration of the protein corona around a spherical gold nanoparticle in a protein-rich environment. Proteins, here shown as blue entangled chains, adhere to the particle's surface forming a ‘hard’ (i) and ‘soft’ (ii) layer. While the inner ‘hard’ layer consists of proteins strongly adsorbed to the particles surface, the outer ‘soft’ layer is formed by weak protein–protein interactions.

A prominent mechanism for aiding cellular uptake *via* endocytotic pathways is to facilitate the diffusion by employing surface modification. Following production by previously discussed synthetic methods, there is a common requirement for stabilisation in order to prevent further growth or aggregation. Charged ligands such as citrate are often utilised for this purpose as coulombic repulsion serves to keep produced NPs separate. However, the negative charge possessed by citrate and other ligands is unfavourable for interaction with the similarly negatively charged cell membrane upon nanoparticle feeding, a process which rather favours small uncharged cargos. In solution-based conditions, macromolecules such as serum proteins adhere to the noble metal surface by physical or chemical adsorption to produce the double layer structure displayed in Fig. 4, known as the ‘protein corona’. These two layers about the colloid consist of an innermost ‘hard’ layer bound directly to the Au surface by strong attractive force and secondary outer layer, comprised of proteins weakly adsorbed to the nanoparticle *via* intermolecular forces such as protein–protein interaction which shields the inner layer from the particle environment.^90^ Despite this, the make-up of the protein corona is subject to dynamic changes in composition and thickness (usually measured between 3–15 nm in thickness) with adsorbed proteins affecting the hydrodynamic diameter of an NP. Initial formation of the corona occurs rapidly upon contact and in the case of culture medium, a stable and biocompatible structure is formed.^90^ This process is of huge synthetic advantage for functionalisation of nanoparticles to achieve facilitated uptake and cellular compartment targeting as even inner layer proteins can be substituted at the surface following a few hours’ incubation with desired ligands, requiring minimal sonication or heat input. This hard corona layer gives the nanoparticle its biochemical identity as experienced by the cell upon contact and remains relatively unaffected by biophysical events such as internalisation- therefore selection of ligand is crucial in achieving the desired uptake and localisation of a SERS nanoprobe. However, environmental changes such as enzymatic presence or the pH are mainly experienced by the outer corona layers, allowing for the use of organic molecules as SERS labels or attachment of fluorescent tags in (or as) the inner layer.

As previously mentioned, surface modification of AgNPs by TATHA2 has achieved enhanced uptake by endocytosis in yeast cells.^54^ Cellular entry and escape from endolysosomal vesicles into the cell through the plasma membrane is associated with the ability to penetrate or pass through the lipid bilayer. Permeability of the lipid bilayers is known to be achieved by the interaction of cationic molecules with the negatively charged plasma membrane which is thereby disrupted and forms nanopores. The choice of molecules spans amine-containing polymers,^6–8^ polypeptides^9^ and cationic liposomes.^10–12^ PEGylation of NPs is a very common surface modification for aiding cellular internalisation, providing a ‘masking’ effect for the NPs to cells of the immune system.^91^ They are also more tolerant to salt aggregation than either citrate or CTAB coated NPs, with smaller cargos being less imposing to cell biology. An additional benefit is that PEG coating hydrophilises the NP surface to serve as a ligand for the attachment of drugs or genes for targeted delivery.^92–94^

Once internalised, a wide range of biochemical delivery strategies are known for evading endolysosomal vesicles. Typically, they are based on the conjugation of the particle surface with various types of molecules such as cationic molecules,^1^ protein transduction domains, CPPs^2–4^ and ligands.^5^ Rhim and co-workers have demonstrated the cytoplasmic localisation of AuNPs conjugated to cationic liposomes alongside plasmid DNA resulting in an improved efficiency of cellular transfection.^95^ This is thought to be achieved by charge neutralisation by liposomes of the plasmid–AuNP complex which results in a more favourable interaction with the plasma membrane.^12^

Furthermore, CPPs have widely been applied to target specific intracellular organelles. Many peptide sequences have been derived from natural systems used by viruses such as the TAT peptide derived from the HIV-1 virus which, conjugated to AuNPs, is known to allow cytoplasmic localisation.^3,13–15^ Even though the exact mechanism of its interaction with biological membranes is not yet known, TAT is thought to mediate cellular import *via* multiple routes including direct membrane passage^14,16^ and intake through endocytosis.^15,17^

Ligand molecules facilitate the directed interaction of the cargo with specific receptors by binding to them. This can be used for the delivery of AuNPs into cells as for instance shown by Tkachenko and co-workers who demonstrated that NPs modified with adenoviral RME peptides gain entry into cells *via* RME.^75^ Furthermore, ligands have particularly been used to target selected cellular components such as the nucleus with attachment of nuclear localisation signal (NLS) peptides.^63,65^

### Toxicity

A vital consideration to the intracellular SERS experiment is the effect of the administered NP on its host. That is, any change or decline in key cellular processes induced by particle–cell interactions decrease the validity of the investigation conducted, as the *in vitro* cell model becomes less representative of the natural state. In general, cytotoxicity of NPs is attributed to the size of both single particles and clusters thereof, surface modifications, NP concentration and composition of the protein corona in addition to the cell type.^24,96–98^ Their size determines the crossing of lipid membranes by NPs into various subcellular structures possessing size exclusive pores. Confinement of NPs in cellular vesicles such as endosomes may however still present toxicity, when the local concentration of the metal far exceeds its LD_50_ (the concentration at which a molecule dose is fatally toxic to 50% of a population). Smaller particles have high surface to volume ratio and therefore can have increased level of interactions with the environment. Indeed single particles of size less than 2 nm in diameter have been shown by several studies to have damaging effects on cells.^96,99–101^

In addition to size based effects, homeostatic mechanisms are also disrupted by the presence of ‘foreign’ metal within the cell. Oxidative stress results from the production of reactive oxidative species (ROS) upon cellular recognition of foreign material and is usually compensated for by homeostatic defence mechanisms. However, at high metal concentrations these mechanisms are unable to maintain normal cell function, therefore cause toxicity and cell death.^100^

Surface modifications also impede upon cell viability. Oxidative stress, evidenced by compromise of mitochondrial potential, integrity and substrate reduction along with presence of ROS, was induced in HeLa cells by triphenylphosphine monosulfonate capped 1.4 nm AuNPs to show increased cytotoxicity relative to 15 nm AuNPs of similar chemistry.^100^ However, pretreatment of the 1.4 nm NPs with reducing agents/antioxidants *N*-acetylcysteine, glutathione, and TPPMS reduced the observed toxicity. AuNPs of similar size but capped with glutathione also induced no oxidative stress. Besides the size dependence, ligand chemistry was also therefore defined as a critical parameter for biocompatibility and toxicity of AuNPs.^100^

The material of the nanoparticle is bound to have an effect. For instance, AgNPs are frequently reported as toxic^102–106^ by the induction of oxidative stress to host cells, exhibiting protein misfolding, mitochondrial dysfunction and impaired DNA repair leading to cell death.^103^ This apparent toxicity can however be advantageous for applications in cancer therapy, with AgNPs shown to be toxic towards tumour cell lines HeLa and U937.^105^ In contrast, AuNPs are usually considered to be non-cytotoxic and inert with limited reports of adverse effects.^104,107–109^ Direct comparison of the two materials’ toxicity is complicated by the large number of factors described in this section, including the differing surface coatings required for stability.

The effects of shape, surface chemistry and NP concentration have been reported by Hutter,^110^ who studied the viability of microglia when incubated with spherical AuNPs, nanorods and nanourchins coated with either PEG or CTAB. No toxicity was associated with any variant at low culture medium NP concentrations (<9 × 10^9^ NPs per mL), however at 9 × 10^9^ NPs per mL cellular activity was reduced by 94% following exposure to CTAB coated nanospheres with no relative toxicity exhibited in the other CTAB particle shapes (slight toxicity in CTAB nanorods with 13% activity reduction at 10^11^ NPs per mL). The modest reduction in cell viability in Au nanospheres compared to Au NRs was likely due to the increased uptake rate of nanospheres. The finding that CTAB coated NPs bore a greater toxicity than those coated with PEG is consistent with numerous other studies.^25,110^ CTAB is used in synthesis as a shape-directing agent and stabilising ligand due to its positive charge, which forms strong interactions with the negatively charged plasma membrane. This disruption of membrane structure is responsible for CTAB's associated toxicity to cells at concentrations over ∼10 nM.^97,101,110^ Reduction of this toxicity can however be achieved by surface modification with other capping agents such as PEG,^111^ PAA,^112^ PAH ^112^ and polystyrene sulfonate.^113^

In spite of numerous toxicity studies, there remains a fundamental requirement for standardisation of investigative procedure before an acceptable consensus on AuNP toxicity can be reached.^114^ Various shortcomings in toxicity assays for NP applications have been identified, including lack of complete characterisation of NPs utilised in experiments, inconsistent reporting of NP concentration (mass per volume gives no indication of NP size, which directly impacts toxicity), and a lack of standardisation of toxicology method parameters such as dose range and incubation media,^115^ all of which make comparisons between nanoparticles complicated. For instance, the aforementioned toxicity of CTAB has also been attributed to the free molecule's presence in AuNP solution, which raises the need for tight ‘supernatant control’ steps to be taken for valid NP toxicity evaluation.^97,112^ Additionally, NPs themselves may affect performance of routine toxicity assays, requiring validation in the presence of the specific type of NP employed as variation in structure or size may alter the performance differently.^115^ Current ambiguities arise from two predominant factors, the first being the complex governance of the phenomenon by a large number of both chemical and physical properties of the NP along with cell types and the degree of NP internalisation. The second being a lack of standardisation in experimental design, execution of procedure and data treatment.^114^

To illustrate the latter point, Fratoddi *et al.* gathered existing data from published studies conducting MTT assay of HeLa cells exposed to differently functionalised AuNPs, for analysis by the metric of numerical particle concentration as opposed to mass concentration or particle size.^114^ This produced a simplified view of how parameters such as NP size, concentration and surface coating impact individual cell viability and concluded that size dependence is much less important than the number of particles present per unit volume. Differently functionalised AuNPs behaved similarly, with the surface coating of the NPs defining the range of particle concentrations at which toxic effects commence.

Thus overall, the requirement for standardisation of nanoparticle internalisation protocols and data treatment remains eminent for the progression of SERS experimentation on single cells *in vitro* and beyond, however promising steps are being taken to achieve this.

## Intracellular SERS

The first application of SERS within cells was carried out in 1991 when Nabiev *et al.* detected the presence of antitumor drugs doxorubicin (DOX) and 4′0-tetrahydropyranyl-adriamycin (THP-ADM) in the nucleus and cytoplasm of living cancer cells using citrate reduced AgNPs.^116^ Since its first intracellular utilisation, SERS has been successfully applied for detection of a vast array of biological markers and metabolites, in turn for sensing and tracking environmental changes through cellular pathways and processes. These include monitoring of cellular functions,^117,118^ dynamics,^119,120^ enzyme kinetics,^121,122^ stress response,^123^ apoptosis^124^ and cell death^125^ along with probing specific compartments such as the mitochondria^126^ and tracking of drugs released into the cytoplasm by NP carriers.^61,127,128^

The cell-based SERS experiment generally takes one of the following two methodologies: the SERS-reporter (SERS-label) approach or the reporter-free (label-free) SERS approach. While the SERS-reporter approach has been more dominant over the last decade the merits of the label-free approach have begun to emerge with concomitant advancements in computational approaches. A review of research advances and developments in both methodologies is presented below. For general and exhaustive reviews of SERS applications in the biological context, also covering the SERS-reporter approach inside cells, the reader is referred to a few excellent recent publications.^5,70,129,130^

### Advances in SERS reporter research

The first and more comprehensively studied intracellular SERS technique is the SERS reporter approach, whereby NPs are functionalised with covalently bound, strongly Raman active organic molecules. Such molecules serve specific sensing purposes by binding to a target molecule. The generated SERS spectrum then reveals the defined signature of the label to facilitate indirect and highly sensitive detection of the target molecule for Raman sensing applications. A specific example of this concept is the use of 4-mercaptobenzoic acid (4-MBA) functionalised NPs as pH sensors, which are sensitive to changes between pH 6–8 within their *in cellulo* environment.^131,132^ This is owed to spectral differences between the molecule's protonated and deprotonated forms (1430 cm^–1^ COO^–^ stretch, specific to deprotonated form in basic conditions).^131^ More recently, this 4-MBA pH sensing was multiplexed with simultaneous measurement of redox potential in live EAhy926 cells. The redox sensitive band at 1666 cm^–1^ (C=P stretch) reports on the oxidation state of SERS reporter molecule *N*-[2-({2-[(9,10-dioxo-9,10-dihydroanthracenyl) formamido]ethyl}disulfanyl)ethyl]-9,10-dioxo-9,10-dihydroanthracene-2-carboxamide (AQ), which was ratiometrically plotted against its redox insensitive 1606 cm^–1^ (C=C stretch) vibrational mode. Cluster analysis then permitted quantitative analysis of redox potential dysregulation, a common indicator for the progressive pathology in neurodegeneration, cardiovascular diseases and cancer.^132^

The density of SERS labels on the surface of the plasmonic NP can be adjusted to experimental needs. For instance, the co-adsorption of other, undesired molecules as well as spectral interference can be avoided by complete coverage of the NP's surface with the label. Additionally, a (further) physical and chemical protection of the probes can be achieved by encapsulation with a silica layer as conceptually introduced by Mulvaney and co-workers^133^ following subsequent advancement in synthesis to achieve ultrathin silica shells.^34,83,134^ Hollow AuNPs can also be protected by PEG capping, offering stabilisation and red shifting their SPR from 700 nm for detection by 785 and 1064 nm irradiation.^135^ Many SERS labels (reporter molecules) have been developed since the technique was established including enzymes, dyes, peptides and DNA which allow for multiplexed detection of numerous molecules using only a single laser wavelength. Multiplexing of SERS reporter functionalised nanoprobes allows for a much broader range of molecules to be simultaneously studied when compared with fluorescence microscopy, due to spectral resolutions of <2 nm which can enable access to between 10–100 unique optical signatures as provided by varying reporter molecules.^65^

**Fig. 5:**
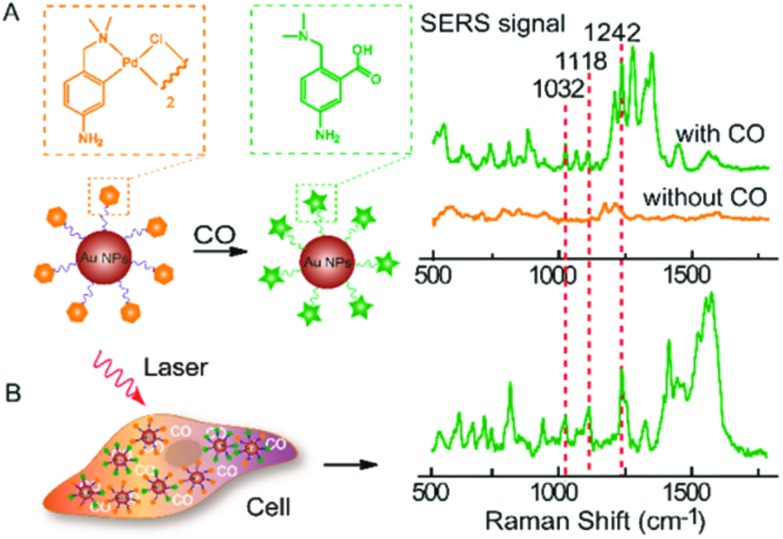
(A) SERS response and sensing mechanism of palladacycle carbonylation on AuNP/PC nanosensors for CO. (B) SERS detection of CO in living cells using AuNP/PC nanosensors. Reprinted with permission from Y. Cao, D.-W. Li, L.-J. Zhao, X.-Y. Liu, X.-M. Cao and Y.-T. Long, *Anal. Chem.*, 2015, **87**, 9696–9701. Copyright (2015) Anal. Chem.

SERS reporter experiments benefit from the specificity of attaching a specific organic molecule to the NP and mapping its known SERS signature, suitable for various intracellular sensing applications. Stevenson *et al.* presented the first use of SERS reporter methodology to monitor the intracellular activity of specific enzymes, a tool which could possibly be implemented to detect dysfunction underlying many diseases.^136^ A recent example of this concept is the detection of carbon monoxide in normal human liver and HeLa cells.^137^ This sensing is based upon the sensitivity of SERS signal from 40 nm AuNP-bound palladacycle reporter ligands to carbonylation, induced by the presence of carbon monoxide, whereby the palladium constituent is removed from the ligand to form a carboxylic acid group as presented in Fig. 5. ^137^ Inside cells, the presence of carbon monoxide releasing molecules (CORMs) was thus evidenced by the appearance of spectral bands at 1032 cm^–1^ (R–NH_2_ rocking), 1118 cm^–1^ (*ν*CX stretch) and 1242 cm^–1^ (*ν*CO stretch). The lowest detection limit of CORMs was found to be 0.5 μM in HeLa cells, attributed to the 1032 cm^–1^ vibrational peak.^137^ This finding offers potential to utilise palladacycle based SERS reporters as an analytical technique to develop a much required knowledge of pathophysiological events involved with carbon monoxide. These examples demonstrate the typical sensing-styled application of intracellular reporter SERS methodology as well as its high sensitivity.

As a well understood technique with high specificity, SERS reporter methodologies have been developed into therapeutic applications such as the detection of inflammation as demonstrated *in vivo* in murine models with twice the sensitivity of two photon fluorescence techniques.^138^ However, SERS is of particular interest to oncology. Antibodies selective to proteins overexpressed in cancer cells have been used to target SERS reporter probes for diagnostic applications.^139–142^ Dual SERS-fluorescence diagnostics have been carried out using a fluorescently labelled aptamer for targeting protein tyrosine kinase, conjugated to Au–Ag nanorods also bound with Raman reporter molecule 4-aminothiophenol. SERS and fluorescent signals could be excited independently using different wavelengths of light for combined cancer cell recognition.^141^ Monoclonal antibodies have also been used to selectively target 40 nm AuNPs with squaraine dye reporter molecules to epidermal growth factor and p16/Ki-67 receptors to achieve selectivity to and recognition of cancer cells’ surface and nucleus.^142^ Therefore, SERS can be presented as a potential means of fast and accurate cancer diagnosis compared to time consuming immunocytochemistry methods.^141,142^

Efforts have been made to combine the detection of cancer biomarkers with microfluidic technologies,^143–147^ however this is mostly conducted at the cellular level. For instance, circulating tumour cells were detected in blood plasma by labelling of Ag–Au nanorods with a mixture of four SERS dyes, along with an ‘antibody rainbow cocktail’ of four breast cancer antibodies and a specific leukocyte CD-45 marker. This resulted in highly specific detection of a single cancer cell in a population of seven million blood cells when applied with multicolour and photothermal imaging. The presence of the SERS reporter dyes could be effectively imaged on the membrane of such tumour cells.^139^ Similarly, multicore SERS reporter labels functionalised with epithelial cell adhesion molecule antibodies have been shown to bind the membrane of living MCF-7 tumour cells without cellular uptake following just 25 ms exposure time. Potential for enumeration and sorting of circulating tumour cells is therefore very promising with microfluidic chips.^147^

**Fig. 6:**
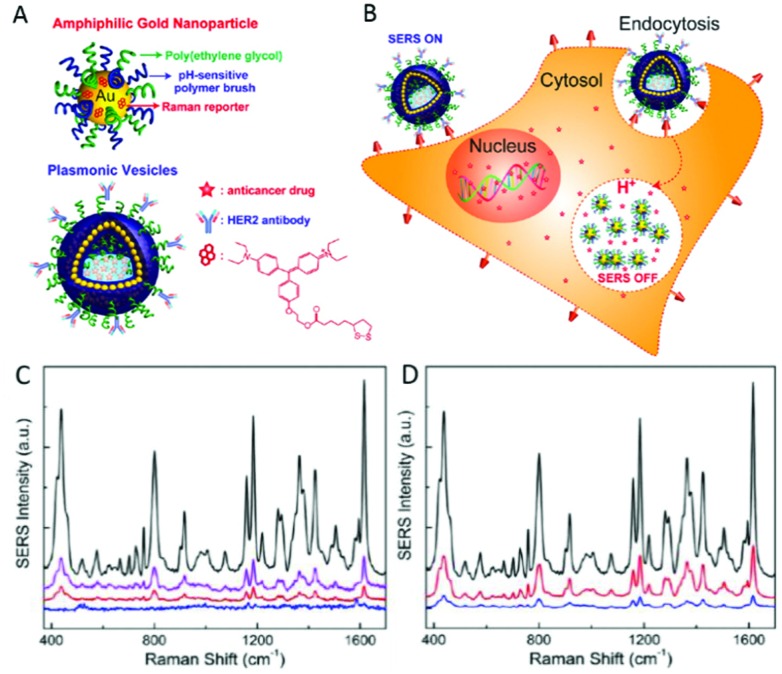
(A) Schematic illustration of the amphiphilic gold nanoparticle coated with Raman reporter BGLA and mixed polymer brushes of hydrophilic PEG and pH-sensitive hydrophobic PMMAVP grafts and the drug-loaded plasmonic vesicle tagged with HER2 antibody for cancer cell targeting. (B) The cellular binding, uptake, and intraorganelle disruption of the SERS-encoded pH-sensitive plasmonic vesicles. (C) Representative SERS spectra of SKBR-3 cells treated with HER2-targeted vesicles (black line) and untargeted vesicle (purple line), MCF-7 cells treated with targeted vesicles (red line), and SKBR-3 control cells (blue line). (D) Representative SERS spectra of SKBR-3 cells labeled with targeted vesicles after 30 min incubation (black line) and the postincubation spectra of the cells at 60 min (red line) and 90 min (blue line). Reprinted with permission from J. Song, J. Zhou and H. Duan, *J. Am. Chem. Soc.*, 2012, **134**, 13458–13469. Copyright 2012 J. Am. Chem. Soc.

Of great relevance to subcellular events is utilising SERS reporter NPs to achieve traceable intracellular drug delivery in therapeutics. A AgNP-loaded, graphene oxide based nanoplatform has been employed by Huang *et al.* to not only achieve pH sensitive delivery of antitumour drug DOX into live cells, but also to monitor its release by SERS.^148^ Similar pH sensitive DOX release and SERS tracking has also been achieved by loading into multi-walled carbon nanotubes decorated with 4-MBA labelled Au and Ag core–shell NPs, verifying the drugs release inside lysosomes by imaging of its fluorescent signal with mapping SERS-determined cellular pH.^149^ Song *et al.* performed successful delivery and monitoring of DOX in live SKBR-3 cells using hollow, amphiphilic SERS-reporter vehicles.^62^ These particles consisted of 14 nm diameter AuNPs, coated with hydrophilic PEG, pH-sensitive hydrophobic PMMAVP grafts and the Raman reporter molecule BGLA (2-(4-(bis(4-(diethylamino)phenyl)(hydroxy)methyl)phenoxy)ethyl 5-(1,2-dithiolan-3-yl)pentanoate). The functionalised AuNPs were then self-assembled into hollow plasmonic vesicles tagged with HER2 antibodies for cancer cell targeting and loaded with DOX as shown in Fig. 6A and B. Upon internalisation of the NP, its degradation is promoted by the pH decrease observed alongside the maturation of endosomes into lysosomes. Not only does degradation release the drug cargo into the cell, but dissociation of the assembled AuNP layer diminishes the previously high SERS reporter signal intensity as the dye is no longer held in SERS hotspots. However, as it is still bound to a single AuNP, signal remains sufficient to probe the chemical environment of the vesicle. While the vesicle-labelled SKBR-3 cells displayed the strong Raman fingerprint of the BGLA probe, significantly weaker signals were detected from cells exposed to non-targeted vesicles and MCF-7 cells incubated with targeted vesicles (Fig. 6C and D). Those labelled with pH-sensitive vesicles also exhibited gradually reduced SERS intensity in the same timeline, as confirmed by the results of plasmonic imaging. Rather than merely detect the reporter compound employed, this approach monitors intracellular drug release based on the loss of hotspot-generated SERS reporter intensity to infer successful drug cargo delivery.^62^ Uncontrolled AuNP aggregation often causes inhomogeneity in SERS measurements (discussed in the next section on reporter-free SERS), yet has been manipulated in this study to yield a novel analysis technique. Although NP-based long term drug delivery has been employed for a significant length of time, the prospect of monitoring the release of the drug intracellularly using reporter SERS represents an exciting development across a range of therapies with suitable antibodies available. In addition to drug release and monitoring, AuNPs have been coated with bifunctional conducting polymer materials, acting as both Raman reporter molecule and a stable surface coating to offer purpose in photothermal therapy. These NPs are capable of highly efficient near-infrared photothermal transduction for cancer therapy whilst possessing good stability and biocompatibility both *in vitro* and *in vivo* in mouse models.^150^

Reporter SERS methodology can also be used to interrogate the intracellular distribution of various molecules by multiplexing the Raman signal of specific reporter molecules to produce SERS map images of single cells. The potential for this was demonstrated by Gregas *et al.*, who targeted 4-MBA-AgNP probes to the cell nucleus with HIV-1 protein derived TAT sequence modification of NPs to characterise their location and produce two dimensional SERS images of PC-3 human prostate cells.^151^ However, uncontrolled aggregation of spherical NPs not only causes locally heterogeneous enhancement of Raman signal by variable SERS hotspot generation, but also may prevent their internalisation or disrupt cell function due to the size of agglomerates. As a result, nanostars may be used for intracellular imaging and sensing. Those bound with nile blue, a dye used as a Raman reporter, and capped with bovine serum albumin have been utilised to map adenocarcinoma A549 and alveolar type II cells to identify constituents such as proteins, nucleic acids, lipids and carbohydrates as well as verifying that the SERS nanoprobes’ primary method of cellular internalisation was *via* endocytosis.^152^ Nanostars of 60–70 nm diameter, labelled with toluidine blue (TB) and encapsulated in a 30 nm coating of silica have also been employed for probing of the intracellular matrix.^153^ The performance of these particles was compared to spherical AuNPs with the same TB reporter and coating. Nanostars were shown by TEM to enter HeLa cells without any tendency to aggregate, albeit at a 3-fold reduction in internalisation. Upon irradiation with an 830 nm laser, SERS signal intensity from the nanostars was mapped across single cells at intensities greatly increased than that generated by spherical NPs, which barely exhibited a response. However, it must be noted when excited at 633 nm, a well-defined SERS signal could also be registered from the spherical nanoparticles, demonstrating the presence of the label within the silica shell.^153^ Therefore the intensity differences could be due to the excitation wavelength and plasmon resonance dependence of enhancements.

Three dimensional SERS elucidation of Chinese hamster ovary cell nuclei was achieved by McAughtrie *et al.* using nanotags functionalised with Raman reporters 4-mercaptopyridine, 5′5-dithiobis(2-nitrobenzoic acid) and 4-nitrobenzenethiol combined with multivariate analysis techniques.^154^ With data trends explained in all three dimensions simultaneously, multiple component detection was possible along with location of internalised nanotags was determined without the need for destructive TEM imaging. Such multi-marker approaches offer importance in delivering full characterisation of disease states or multiple cell organelle targeting while three dimensional analysis increases validity in modelling complex cellular structures.^154^

The probing of the intracellular chemical environment is very much a key application for SERS research given its high resolution and non-destructive nature. The ability to sensitively detect, map, monitor and quantify the localisation of both endogenous and extraneous molecules at the sub-cellular level would unlock a world of new information regarding the molecular changes undertaken by live cells during a range of cellular processes. The SERS reporter methods presented here offer promising steps towards visualisation and quantification of targeted molecules across individual cells. However, the effects of vast amounts and variety of biomolecules surrounding the nanoprobes go unaccounted for, limiting the amount of detailed information available as molecular events unfold. Despite the ability to multiplex data with numerous reporter molecules, this is a limitation of the technique when fully characterising cellular pathways. The approach may also lack spectral resolution in some applications where characteristic reporter bands (such as the phenyl ring modes of 4-MBA) only exhibit small wavenumber shifts (few cm^–1^) as a function of bound substituents.^155–158^

### Advances in reporter-free SERS

In contrast, in the reporter-free SERS approach the nanoparticles probe their direct vicinity and thus can potentially sense the large complexity of the chemical environment around them. In reality the affinity of molecules to the surface chemistry (charge) will determine the environment in the direct vicinity of plasmonic particles (*e.g.* AuNPs). Molecules near or adsorbed onto an NP or into a NP's protein corona determine the measured SERS spectrum and allow for (bio)chemical sensing and characterisation, to track cellular processes such as cellular differentiation by DNA/RNA ratios, for instance.^63^ As mentioned earlier, single NPs can aggregate, giving rise to regions of intense enhancements called hotspots between the particles. This electromagnetic field enhancement between close-proximity SERS probes causes molecules located in those hotspots to provide a larger contribution to the measured SERS spectrum, albeit non-uniformly, as the aggregation and hence the generation of hotspots inside cells is largely uncontrolled. While this makes quantitative SERS measurements difficult, it allows for the qualitative but highly sensitive detection of the molecular environment inside cells.^159^

Despite the simple NP preparation involved in reporter-free SERS, selectively identifying molecular changes resulting from cellular processes in large and multidimensional data sets (arising from the large variety of biochemical molecules detected) can be particularly challenging especially in the more complex, eukaryotic (including mammalian) cells. Although examples of specific detection of molecules, wherein metabolites such as FAD and FMN have been identified in bacterial cells,^160^ with continued development of reporter-free intracellular SERS applications with eukaryotic cells are likely to emerge. Moreover, the lack of simple tools for extracting this information has so far resulted in limited characterization of fundamental cellular processes, especially, by reporter-free SERS approaches. However, when applied effectively, this allows access to the vast wealth of information available from the raw SERS data acquired from biological samples such as cells.^64^

Owing to the fact that this approach samples numerous constituents of the chemical environment around a NP, reporter-free SERS studies have been guided towards tracking and characterisation applications. In a similar technique outlined with SERS reporter methodology,^62,148^ the release and delivery of anticancer drug DOX was specifically tracked within live human oral squamous carcinoma (HSC-3) cells.^61^ AuNPs of 28 ± 3 nm diameter were functionalised with NLS and RGD peptides following (PEG)ylation, with the DOX bound to the Au surface by Ph-sensitive hydrazine linkage. Upon pH-dependent release of the drug within lysosomes, the intensity of its SERS band at 460 cm^–1^ was diminished with release over a 12 h period, with its inherent fluorescent signal no longer quenched by a close-proximity AuNP used to verify DOX localisation in lysosomes and resulting release into cytoplasm.^61^

The Kneipp group carried out an early probing of cellular compartments by reporter-free SERS in living macrophages and endothelial cells. Differences in the SERS spectra obtained in different cell lines and over time, as well as the direct identification of physiologically relevant molecules, demonstrated that reporter-free SERS approaches are feasible for the characterisation of changing cellular environments and useful for intracellular applications. Additionally, the tendency of internalised AuNPs to uncontrollably aggregate results from the changing chemical environment experienced by the probes, with dimer and trimer formation (revealed by Transmission Electron Microscopy, TEM) leading to increased enhancement of the Raman signal compared to individual particles.^16^ Thus, controlling or even preventing this aggregation of NPs inside the cell in order to eliminate the incurred heterogeneity of acquired spectra from hotspots remains one of the key challenges to reporter-free SERS experiments inside cells. Novel structures, which show high plasmonic enhancements at the single nanoparticle level compared to single spherical nanoparticles, have been employed to overcome this hurdle, as are discussed later in this section.

Reporter-free SERS for mapping the intracellular environment has nevertheless been used in many applications. In a study conducted by Zhang *et al.* a microfluidic chip was used for delivery and immobilisation of single cells.^161^ They used ordered continuous flow to expose Chinese hamster ovary (CHO) K1 cells to intracellular flux agonist ionomycin. Following mapping to reveal the location of the cell nucleus, cytoplasm and membrane, the approach was found capable of monitoring the chemical changes occurring within the cell during ionomycin-evoked Ca^2+^ flux response. This was achieved by observing the time profile of changing amide I (1643 cm^–1^) concentration, showing a similar trend to that of Ca^2+^ flux which is commonly used in biomedical research to stimulate the intracellular production of proteins such as interferons.^162^ This demonstrates the potential to utilise SERS for *in situ* monitoring of molecules to characterise protein expression dynamics at subcellular levels. The continuous flow methodology developed also permits more time effective experimentation when a series of SERS measurements are required, relative to batch preparation and analysis.^161^

Reporter-free SERS has also demonstrated the capability to differentiate between closely related cell phenotypes in progenitor and differentiated cell (DC) types.^63^ The SV-40 large T nuclear localisation signal peptide, containing a fluorescein (flu) tag at its C terminus for confirmation of attachment, was bound to 40 nm AuNPs *via* a cysteine linker and used to probe SH-SY5Y cells with 633 nm excitation, producing cellular maps at a resolution of 200 × 600 nm per pixel. Unambiguous characterisation of DC and undifferentiated cells (UDCs) was achieved through principal component analysis (PCA) across both the whole cell and the nuclear region. Furthermore, characterisation of cell differentiation was carried out by examination of nuclear regions, which undergo changes in ratios of molecular content as well as morphological and structural development.^163^ Reorganisation of chromatin plays an important role in the proliferation of non-dividing cells (DCs) which maintain a steady molecular formation relative to UDCs whose nuclear regions undergoes continuous changes in chromatin formation during mitosis. The acquired SERS data revealed a shift towards increased ratio of DNA/RNA in DCs relative to UDCs, as revealed by respective characteristic peak intensities. The number of protein peaks detected in DCs was also increased, indicating greater expression and variety of protein within the nuclear region.^63^ This characterisation of cellular differentiation exhibits not only the ability of reporter-free SERS to distinguish between cell phenotypes but also how the careful application of statistical analysis methods such as PCA are utilised to effectively reduce the complex data matrices to unambiguously achieve classification.

**Fig. 7:**
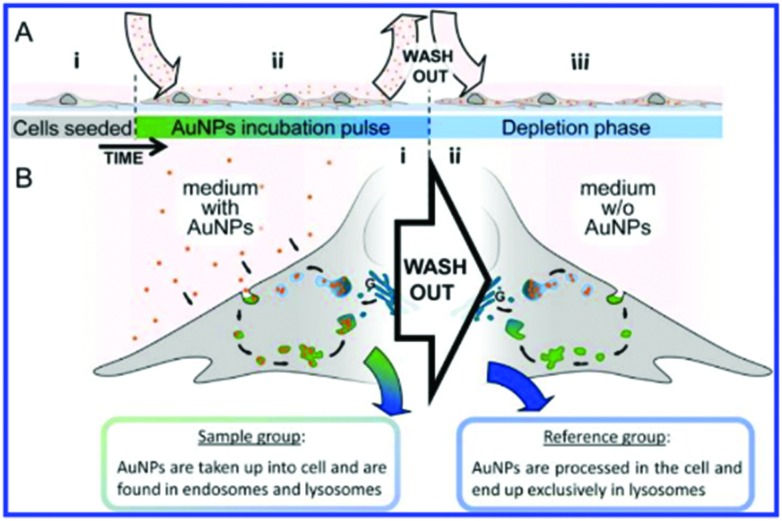
Schematic of experimental design. (A) Full experimental procedure involves AuNPs (red spheres) being added to the cell culture environment after (A.i) cells have sufficiently attached to the culture dish. (A.ii) Following uptake of the particles into the cell *via* endocytosis during the incubation pulse, extracellular particles were washed out. (A.iii) Fresh culture medium without AuNPs was added and the cells were left until incorporated particles were processed into lysosomes (depletion phase). (B.i) During the incubation phase, cells constantly internalize AuNPs, which accumulate inside endosomes (green vesicles) and lysosomes (blue vesicles), their acquired SERS map data serves as the sample group for analysis. (B.ii) Following the wash-out, vesicular AuNPs are processed along the endolysosomal pathway and are eventually found exclusively in lysosomes. SERS maps of cells with only lysosomal AuNPs serve as the reference group for the data analysis. Reprinted with permission from A. Huefner, W.-L. Kuan, K. H. Müller, J. N. Skepper, R. A. Barker and S. Mahajan, *ACS Nano*, 2015. Copyright 2015 ACS Nano.

**Fig. 8:**
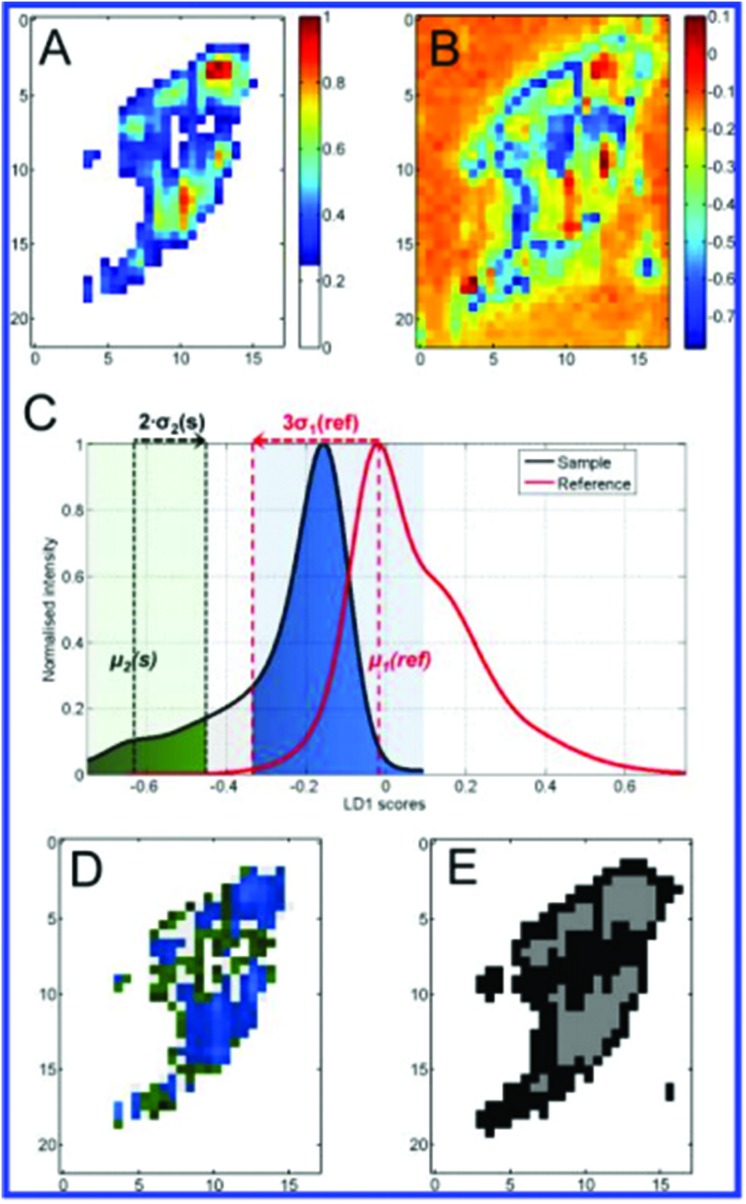
Demonstration with a test sample cell of different analysis steps and comparisons. Bright-field image (A) highlights the scanned area resulting in the corresponding absolute SERS intensity map (B). Pseudo colour PC1 intensity map (C) of the same cell after PC analysis shows the sample background as white for PC1 scores values smaller 0.25. LD1 scores map (D) of the sample cell prior to endosomes/lysosomes classification. (E) Assignment of components from the LD1 scores intensity distribution to either lysosomes (LD1(s) > –0.35) or endosomes (LD1(s) < –0.45) highlighted in blue and green, respectively. Assignments were made with respect to the modes μ1(ref) and μ2(s) as well as the standard deviations σ1(ref) and σ2(s) marked as dotted lines. The light grey region between the green and blue regions is a transition region to guarantee an accurate classification. (F) Based on scores for respective class assignments derived from (E), scores were projected back into a false-colour map. This generates a color-coded reference-based PCA-LDA map showing endosomes (green) and lysosomes (blue). (G) A colour map was reconstructed after *k*-means clustering of the test data set for the three groups (white, grey, black) confirming the group segregation facilitated using our color-coded reference-based PCA-LDA method. Scale bars: 10 μm, (C, D) and (F, G) pixel size: 600 nm × 600 nm. Reprinted with permission from A. Huefner, W.-L. Kuan, K. H. Müller, J. N. Skepper, R. A. Barker and S. Mahajan, *ACS Nano*, 2015. Copyright 2015 ACS Nano.

More recent work by the same group further effectively demonstrated chemometric analysis in combination with tailored sample preparation to achieve detailed hyperspectral characterisation of endosomes and lysosomes in the endolysosomal pathway in SH-SY5Y cells.^64^ The novel method for creation of a reference state depicted in Fig. 7 was derived from a conventional pulse-chase technique,^16,67^ whereby cells were subjected to a 72 h AuNP incubation pulse to ensure a wide distribution of the AuNPs within various types of vesicle and achieve high SERS signal. Cells analysed in this state were assigned as the *sample group* (*n* = 20). A *reference state* (*n* = 14) was generated by carrying out the same incubation phase followed by a 48 h depletion phase in which the extracellular fluid was removed and replaced with fresh, AuNP-free media to ensure that the nanoprobes were processed through the endolysosomal system and only localised within lysosomes. Background subtraction of the generated SERS map data with principal component (linear summations of original data multiplied by a coefficient that describes the generated principle components, PCs) scores lower than 25% of the maximal PC1 value (Fig. 8A). PCA-LDA (Linear Discriminant Analysis) allows for the transformation of PC data to achieve maximum class segregation by hyperspectral peak positions, ratios and intensities. This was employed to assign PCA-LDA scores (Fig. 8C) as lysosomes (blue, scores common to both sets) or endosomes (green, scores exclusive to the *reference state*) with borderline case exclusion. Exemplarily, the back-projected pseudo colour map of the LD scores (Fig. 8D) clearly depicted the localisation of each vesicle type in a single cell, showing broad agreement with the supervised, multivariate analysis technique *k*-means clustering (Fig. 8E).

Furthermore, characterisation of endosomes and lysosomes was achieved by observation of PC1 loadings, which were weighted with individual peak probabilities to reveal subtle differences in molecular content. These variations identified molecular processes occurring along the endolysosomal pathway as the breakdown of proteins and lipids, progressively raised acidity, and degradation of DNA/RNA in lysosomes.^64^

The avenues by which the successful implementation of this tailored preparation and reference-based PCA-LDA based method address the shortcomings of reporter-free SERS experiments are multifaceted. Firstly, the technique not only allows for extraction of useful data from the complex and large original dataset, but also provides an improvement on existing analysis methods such as standard PCA-LDA (Fig. 8B) and the *k*-means clustering used for validation in this study. Creation of the reference state provides a physical meaning to classes, and hence spectral assignments, whereas *k*-means itself does not allow unambiguous assignment of clusters to specific classes such as endosomes and lysosomes. The method was also verified on negative controls (cells with lysosomes only) and the classification was not only accurate but also demonstrated an advantage over single frequency SERS and simple LD score maps. Mean centring and PCA steps also address the issue of heterogeneous SERS enhancements by uncontrollable NP aggregation.^64^

Intracellular and reporter-free SERS has also been utilised in elucidating the biomolecular dynamics of the stress response to UV-C (254 nm) irradiation in HSC-3 cells.^164^ NLS and RGD targeted Au nanocubes observed damage to cytosolic proteins containing sulphur and aromatic amino acids and changes to their secondary structure. The 502 cm^–1^ disulfide vibrational mode exhibited decreased intensity compared to that of the C–S 653 cm^–1^ mode during irradiation which reflected the photolytic cleavage of disulphide bonds. The UV-C exposure was also determined to completely arrest NP transport within living cells and induce apoptosis, hallmarked by intense vibrations at 1000 and 1584 cm^–1^. Real-time evaluation of defence mechanisms of cancerous cells towards UV exposure is a valuable application of reporter-free SERS in forwarding photothermal therapies.^164^

As mentioned earlier, the uniformity of SERS signals is extremely significant for reporter-free SERS, in particular for quantitative analysis. Considerable efforts have been made into counteracting the uncontrollable aggregation of internalised AuNPs by adaptation and modification of the particle structure. The production of gold lace nanoshells not only generates high SERS enhancements in the 1–3 nm hotspots between branch like structures (3–5 nm) without aggregation, but also offers further application as a model for the delivery and monitoring of hydrophobic drugs.^85^ The topology generated by surfactant mediated production from polyurethanes (PUs) is similar to that of a Faberge egg, with the hydrophobic core (used to cargo drugs) provided within the amphiphilic structure of PU globules. SERS monitoring of loaded pyrene was carried out, the release of which was shown to plateau out after 30 h. The lace nanoshells offer tighter synthetic control of diameter relative to thermosensitive polymer gold cages^88^ and liposome–gold nanocontainers^86^ also used for hydrophobic drug delivery. This aids avoidance of first capture by macrophages and renal clearance when carriers are too large or small respectively. The improved homogeneity of hotspots offers advantage in generating reproducible reporter-free SERS measurements over spherical NPs.^85^

**Fig. 9:**
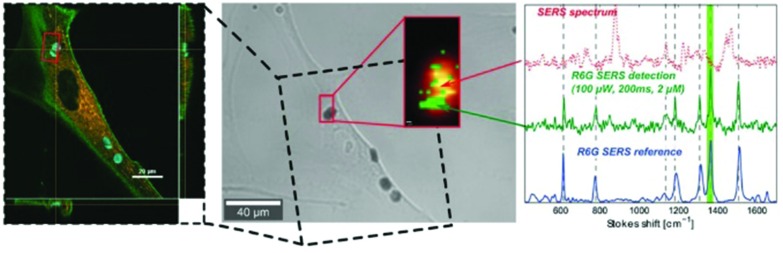
Reporter-free intracellular detection of rhodamine-6G. (A) Confocal fluorescence scan of a single cell with several nanodome-patterned microchips show the intracellular localisation of the microchips, as well as the uptake and inhomogeneous distribution of R6G in the cell. (B) Transmission image of the same cell, where SERS spectra are mapped over the region marked with a red square. The inset shows the integrated number of counts in the 400–1700 cm^–1^ region (graded red-yellow), on top of which the presence of the 1365 cm^–1^ R6G-peak is highlighted in green, which corresponds to the green shaded area in (C). (C) SERS spectra on different positions of the microchip show the intracellular SERS detection of R6G (middle, green) and the R6G reference SERS spectrum (bottom, blue), while other positions show the presence of peaks related to other molecules adsorbed on the microchip (dotted red, top). Spectra are normalized and offset for clarity. Reprinted with permission from P. C. Wuytens, A. Z. Subramanian, W. H. De Vos, A. G. Skirtach and R. Baets, *Analyst*, 2015 – Published by The Royal Society of Chemistry.

Similarly, the top-down construction of gold nanostructured microchips proposes an alternative means of removing the variability inherent to reporter-free SERS by aggregating spherical NPs. Chips with a uniform and reproducible nanodome gold pattern were generated by nanosphere lithography, depositing a gold nanofilm on a monolayer of polystyrene bead templates and thereafter released into solution.^87^ Like spherical NPs, the microchips were taken up by normal human dermal fibroblast (NHDF) cells with no suggestion of reduced viability from observation of mitotic events. A proof-of-concept detection experiment was then carried out using the common extraneous SERS molecule, rhodamine 6G (R6G). Following a 24 h incubation period, the intracellular location of the microchips and inhomogeneous distribution of R6G were confirmed in NHDF cells by confocal reflection and fluorescence microscopy (Fig. 9). The transmission image reveals the location of the cell which was mapped for the 1365 cm^–1^ R6G SERS peak (imaged in green).

This successful detection of a well-established SERS-active compound using nanostructured microchips paves the way for detection of extraneous molecules within live cells with improved reproducibility due to relative homogeneity of hotspot enhancements. This study also partially addressed a previous difficulty in determination of the exact location of internalised AuNPs, given that voluntarily internalised NPs exist in membrane-bound transport vesicles from which they may or may not escape to be free in the cytosol. A lack of co-localisation of the microchips with fluorescent dyes LysoTracker Red DND-99 and Vybrant Dil (accumulating in acidic cellular components and lipophilic membranes respectively) suggested no localisation within vesicles of the endocytotic pathway, although further studies are required to verify exact location. Despite protein corona adsorption to the gold plated chips forming an extra barrier for molecules to cross to reach plasmonic hotspots, this novel and reporter-free approach takes positive steps towards homogenously enhanced, time-dependent and quantitative detection of intracellular molecules by SERS.^87^

Recently, in a powerful combination, reporter-free SERS and SERS reporter nanoprobes were employed by Chen *et al.* to simultaneously image the nucleus and plasma membrane of single HeLa cells in three dimensions, afforded by confocal SERS microscopy.^65^ Characterisation of the nucleus and tracking of time dependent changes in molecular constitution during induced apoptosis was achieved using NLS-functionalised, reporter-free AuNPs. Consequently, slow condensation and degradation of chromatin, nuclear rupture and denaturation and degradation of proteins were all identified. AuNP-bound 4-MBA, Crystal Violet and Cresyl Violet acetate were used as SERS reporters to monitor the localisation and detachment of folate and luteinizing hormone-releasing hormone receptors in real time. The SERS reporter portion of this investigation provided verification that specific endogenous molecules are being monitored, which is sometimes an ambiguity in purely reporter-free studies. Although a fairly laborious process, the combination of reporter and reporter-free SERS techniques displays great promise for *in situ* characterisation of complex intracellular processes, with application of suitable chemometric analysis.^65^

## Conclusions and outlook

As a non-destructive, highly sensitive technique capable of sub-micron imaging resolutions, SERS holds immense potential for interrogation of the intracellular environment across biomedical research. Acquisition times can be optimised down to the sub-millisecond range, sufficient for even single molecule detection.^11,13,165^ Under appropriate laser powers, absence of signal deterioration effects such as photobleaching of samples allows for live cell investigations. Despite a requirement for standardisation of toxicity studies,^114^ the employed nanostructures – particularly those of gold – appear not to impinge upon the viability of live cells. Intracellular SERS studies are still in their infancy; however, this is a fast-paced field attracting high levels of interest with an ever-growing body of research. Advances in nanotechnology have led to an increasingly varied library of SERS active structures, tailored to addressing the difficulties of *in cellulo* experiments such as cellular entry, pH dependent aggregation and targeting of specific compartments.

It is clear that the choice of intracellular SERS experiment performed is very dependent on not only the studied system but also the data required. SERS reporter methodology benefits greatly from simplicity of data analysis, and holds advantage over fluorescence microscopy in increased spectral resolution to allow multiplexing of a larger number of unique optical signatures. The discussed studies offer reporter-based SERS as an effective tool for the imaging of molecules and proteins with known vibrational signatures, as well as relatively simple quantification of simple cellular processes.^131,132,137^ The approach has matured to therapeutic application with multipurpose nanostructures which not only detect and monitor but deliver a cargo of hydrophobic drug molecules into intracellular compartments.^62^

Despite being less widely developed; reporter-free SERS is more suited as a powerful means of probing complex cellular processes due to the sheer wealth of information which can be extracted from a single measurement of the intracellular matrix about the vicinity of the NP. This however comes at the cost of throughput times, as careful processing and analysis of experimental data must be employed to extract desired information in order to accurately and unambiguously characterise processes at the molecular level. Such methodology was effectively achieved in the cases of endocytosis and apoptosis.^64,65^ Combination of reporter-free and SERS reporter techniques yields comprehensive findings, tracking both the localisation of specific proteins as well as monitoring changes in the diverse chemical environment. Tailoring of sample preparation to create a sample set upon which to train multivariate analysis methods such as PCA-LDA prove equally capable with the potentially complex synthesis of multiple functionalised SERS reporter probes. In either case, it remains that the development of statistical methodologies and data analysis toolkits are central to potentiating the use of reporter-free SERS to deconvolute the molecular dynamics of intracellular structures, pathways and processes.

Future developments in intracellular SERS will be of particular interest to the pharmaceutical industry, for elucidating new drug targets in both healthy and pathogenic cells through cell-based assays. Additionally, there exists a requirement for determination of administered small molecules concentration and localisation at the sub cellular level, which no established technology can currently satisfy.^166^ Without information on whether candidates are entering cells, let alone localising to and interacting with target organelles or biomolecules, pharmacodynamics cannot be fully understood which contributes to the lack of *in vivo* efficacy responsible for the failure of a large portion of clinical trials.^166^ Microfluidics, both for nanoprobe synthesis and cellular interrogation will also play a major role in the development of tightly controlled, reproducible and ultimately higher throughput techniques. Along with early-stage diagnostics by SERS-reporter and reporter-free SERS methodologies, these applications should form central goals for future proof-of-concept, assay development and process optimisation studies in order to further develop and implement highly powerful intracellular SERS techniques for interrogation of the cellular environment.
